# The Fundamental Comparison of Zn–2Mg and Mg–4Y–3RE Alloys as a Perspective Biodegradable Materials

**DOI:** 10.3390/ma12223745

**Published:** 2019-11-13

**Authors:** Jiří Kubásek, Drahomír Dvorský, Jiří Šedý, Šárka Msallamová, Jitka Levorová, René Foltán, Dalibor Vojtěch

**Affiliations:** 1 Department of Metals and Corrosion Engineering, Institute of Chemical Technology, 16628 Prague, Czech Republic; Kubasekj@vscht.cz (J.K.); dvorskyd@vscht.cz (D.D.); msallams@vscht.cz (Š.M.); 2 Department of Normal Anatomy, Faculty of Medicine, Palacký University Olomouc, 775 15 Olomouc, Czech Republic; jirisedy@jirisedy.cz; 3 Department of Oral and Maxillofacial Surgery, First Faculty of Medicine, Charles University, 128 01 Prague, Czech Republic; jitka.levorova@gmail.com (J.L.); Rene.Foltan@vfn.cz (R.F.)

**Keywords:** magnesium, zinc, biodegradable materials, in-vivo tests, corrosion

## Abstract

Biodegradable materials are of interest for temporary medical implants like stents for restoring damaged blood vessels, plates, screws, nails for fixing fractured bones. In the present paper new biodegradable Zn–2Mg alloy prepared by conventional casting and hot extrusion was tested in in vitro and in vivo conditions. Structure characterization and mechanical properties in tension and compression have been evaluated. For in vivo tests, hemispherical implants were placed into a rat cranium. Visual observation of the living animals, an inspection of implant location and computed tomography CT imaging 12 weeks after implantation were performed. Extracted implants were studied using scanning electron microscopy (SEM) on perpendicular cuts through corrosion products. The behaviour of zinc alloy both in in vitro and in vivo conditions was compared with commercially used Mg-based alloy (Mg–4Y–3RE) prepared by conventional casting and hot extrusion. Both compressive and tensile yield strengths of Zn and Mg-based alloys were similar; however, the brittleness of Mg–4Y–3RE was lower. Zn and Mg-based implants have no adverse effects on the behaviour or physical condition of rats. Moreover, gas bubbles and the inflammatory reaction of the living tissue were not detected after the 12-week period.

## 1. Introduction

Biodegradable metals and alloys have attracted great attention in the past few decades as promising alternatives to permanent implant materials, like Ti alloys, Co alloys or stainless steels, and to biodegradable polymeric materials, such as poly-lactic acid (PLLA). Biodegradable materials are of interest for temporary medical implants like stents for restoring damaged blood vessels, plates, screws, nails for fixing fractured bones. Although the biodegradable polymeric materials can be used for coronary stent applications, the main problem is related to the load-bearing applications, where polymeric materials often fail because materials available at present suffer from a low hardness, strength, fracture toughness and wear resistance even when additions of ceramic particles are used. When using biodegradable material, instead of a permanent one, the second surgery for removing the implant is not needed after healing which reduces the morbidity, health costs, and inconvenience to the patient. Therefore, great attention is devoted to metallic biodegradable materials, which appear more suitable for load-bearing applications.

Three metals, magnesium, iron and zinc, have been mostly studied up to date concerning the design of biodegradable medical implants because they are relatively non-toxic and essential for proper biological functions of the human body. In the case of magnesium, the research activities lasting more than one century have provided a huge amount of data related to mechanical properties, in vitro bio-corrosion behaviour and in vivo biocompatibility of these materials [1,2,3,4,5,6,7,8,9,10,11,12]. But problems with excessive in vivo bio-corrosion rates of Mg alloys associated with the evolution of undesirable corrosion products have precluded the widespread use of Mg in the medical field. Although commercial magnesium screws have been manufactured from Magnezix (MgYREZr alloy) [13], the actual range of use in medicine is not known. Iron also offers a potential for applications in biodegradable implants. The main advantage of Fe over Mg is a higher strength, but the ferromagnetic behaviour and insufficient in vivo bio-corrosion rates of Fe remain essential topics for further experimental research [14,15,16]. The insufficiently low corrosion rate of pure Fe can be partially increased by alloying with Mn, Pd, W, Sn, B, C, S or Si [17,18], however, in the case of Mn or Si addition, this can lead to the decrease of the viability of a specific type of cells, such as human endothelial cells (ECV304) [18,19,20].

There is currently only a limited number of research works dealing with in vivo assays of degradable stents made of iron [21,22,23,24,25]. These studies did not show adverse effect in tissues of rabbits and minipigs (thrombosis, necrosis, inflammation). Mueller et al. [26] studied the behaviour of iron sheets in the tails of mice. Histological examination showed slow degradation of Fe and accumulation of deposits in some places. This phenomenon has been accompanied by an inflammatory reaction but no indication of toxic effects [26].

Zinc and its alloys have been subjects of biomaterial engineering research for the last few years [15,27,28,29,30] and significant progress and huge interest in the research areas has been recorded recently [30,31,32,33,34,35]. A clear advantage of zinc and its alloys over magnesium is processing. Casting is generally performed in an air atmosphere at lower melting temperatures of about 500–600 °C. Magnesium alloys are melted at 700–800 °C in a protective atmosphere. Besides, zinc-based alloys are characterized by good machinability and can be easily processed by conventional extrusion.

Zinc exhibits similar mechanical properties like magnesium, but in vitro corrosion of zinc is slower in comparison with magnesium [36]. Another beneficial feature of zinc is the absence of hydrogen evolution in the corrosion process [36]. From the biological point of view, zinc is an essential element for biological functions of the human body. It is involved in various aspects of cellular metabolism, supports proper functions of numerous enzymes, immune system, protein and DNA synthesis, normal growth, wound healing, a proper sense of taste and smell [37]. The recommended dietary allowance and recommended upper limit for zinc are 15 and 40 mg per day, respectively [38], but consumption in amounts higher than these values is generally considered relatively non-toxic. Even amounts approaching 100 mg/day can be tolerated for some time [28,38].

The use of pure Zn as biodegradable material is limited due to its insufficient strength, plasticity and hardness, but this may be easily overcome by using suitable alloys.

In vitro biocompatibility investigation of zinc alloys including cytotoxicity tests in the alloy, extracts revealed that zinc is slightly less biocompatible to U-2 Os and L929 cells than magnesium [32,35]. Decreased viability (about 65%) of VSMC cells in alloy extract was observed by Li et al. [33], although the viability of ECV304 and MC63 cells was determined as excellent in the same study. Murni et al. [30] observed generally very good in vitro biocompatibility of Zn-3Mg alloy although decreased viability of NHOst primary cells at specific conditions has been observed. Also, genotoxicity and mutagenicity tests did not indicate any negative effects connected with the use of zinc [30]. In vitro study of pure Zn in which the authors pointed out slightly lower mechanical and cytotoxicity properties for Zn compared to the Mg was performed by Liu at.al [34]. Although the number of in vivo biocompatibility tests with zinc or zinc alloys is still limited [28,33,39,40,41,42], these tests indicate that zinc is a promising biodegradable material. In the study of Zberg et al. [39] an amorphous Mg–Zn–Ca alloy containing about 50 wt % of Zn was prepared by copper-mould-injection casting. The amorphous alloy was then implanted into the abdominal cavity of domestic pigs. It was shown by histological analysis that the implants exhibited the same good tissue compatibility as pure Mg implants, but strongly reduced hydrogen evolution due to the presence of zinc. One drawback of amorphous alloys is a quite difficult preparation procedure requiring high cooling rates and, therefore, small (a few mm in thickness) amorphous samples can only be prepared. Samples of larger volumes prepared at lower cooling rates would be brittle and unsuitable for load-bearing applications, because of the presence of Mg + MgZn eutectic mixture in the structure. The in vivo study of pure zinc was reported by Bowen et al. [28]. They applied thin zinc wires to the abdominal aorta of adult rats and studied corrosion rates, corrosion products and tissue adherence. It was observed that the in vivo corrosion rate ranged between 10 and 50 µm/year and that it progressively increased during six months’ exposition. Another important finding was that the implant remained intact for four months after implantation. Corrosion products on the surface were compact facilitating a good adherence of the surrounding tissue. Bowen et al. [40] and Yang et al. [43] reported excellent biocompatibility, without severe inflammation, platelet aggregation or thrombosis formation on zinc wires or stents in the aorta of rats and rabbits, respectively.

Besides the stent applications, zinc biodegradable alloys are also being explored as potential alternatives to titanium or stainless steels for fixation devices of fractured bones in traumatology. In vivo study of Zn–1Mg alloy in different states has been performed by Li at.al [33]. These tests confirmed the suitability of binary alloys for orthopaedic application, as it guaranteed sufficient mechanical support during the tissue-repair process.

Other novel information considering specific challenges related to zinc is summarized in several review works [44,45,46,47,48].

The present paper deals with the structure, mechanical properties, and in vitro and in vivo corrosion of biodegradable Zn–2Mg alloy. The magnesium addition to the experimental alloy was aimed to increase the strength and hardness, improve biocompatibility, support bone growth and slow down the corrosion rate of zinc [36,49,50]. The commercial Mg–4Y–3RE (WE43, according to the ASTM B93 designation) biodegradable alloy [51,52,53] was also explored as reference material. A similar alloy called Magnezix has recently been approved for clinical testing and use in human medicine [54].

## 2. Materials and Methods

The Zn–2Mg and Mg–4Y–3RE alloys were investigated in this study. Chemical compositions are given in Table 1. The Zn–2Mg alloy was prepared by melting pure metals (99.9% purity) in a resistance furnace under argon atmosphere. The temperature of the melt was kept at 500 °C to avoid excessive evaporation of zinc and oxidation of magnesium. After sufficient homogenization of the melt, it was poured to a cast-iron mould to prepare cylindrical ingots of 20 mm in diameter and 200 mm in length. The Mg–4Y–3RE alloy ingots of 40 × 80 × 500 mm^3^ in size were purchased from an industrial supplier (Luxfer MEL Technologies, Manchester, UK). The alloy was re-melted in a vacuum induction furnace and cast to ingots of the same dimensions as those of the previous material. Before extrusion the Mg–4Y–3RE alloy was heat treated at 525 °C for 8 h. The following step was a hot extrusion of Zn–2Mg and Mg–4Y–3RE conducted at a temperature of 300 °C and 400 °C respectively, extrusion rate of 5 mm/min and extrusion ratio of 10:1 to produce rods of 6 mm in diameter and 80 mm in length.

### 2.1. Structure and Mechanical Properties

**Figure 1:**
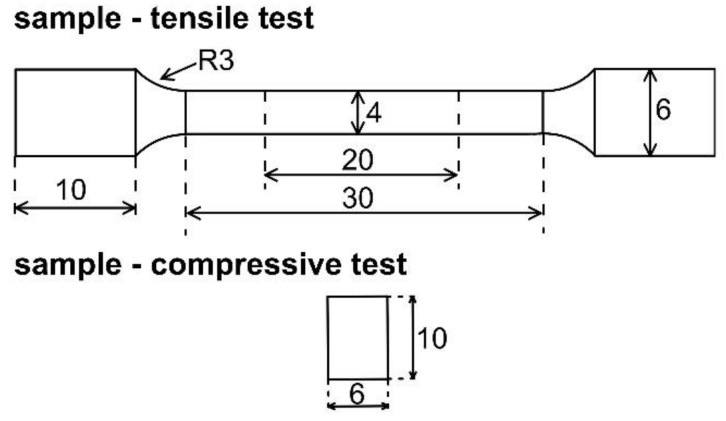
Shape of the samples for tensile and compressive test. All dimensions are in mm.

The microstructures of the alloys were examined by optical (OM) and scanning electron microscopy (SEM, Tescan Vega 3, TESCAN Brno, s.r.o., Brno, Czech Republic) equipped with energy dispersion spectrometry (Oxford Instruments Aztec, Abingdon, UK). For this purpose, the samples were ground using SiC abrasive papers (P180–P4000), polished by diamond pastes with 2 and 0.7 μm particles and etched in a 2 mL HNO_3_ + 100 mL H_2_O solution. The phase composition of alloys was determined by X-ray diffraction (X’Pert Philips, Malvern, UK, 30 mA, 40 kV, X-ray radiation Cu Kα). Mechanical properties of alloys were examined by tensile testing. Tensile tests were performed according to the ČSN EN ISO 6892-1 on cylindrical “dog bone” samples with 4 mm in diameter of gauge length (Figure 1). Tests were carried out on a LabTest 5.250SP1-VM universal loading machine (LABORTECH s.r.o., Opava, Czech Republic) at a deformation rate of 1 mm/min. Compressive tests were performed according to the ASTM E9–19 (Standard Test Methods of Compression Testing of Metallic Materials at Room Temperature). The cylindrical samples with 6 mm in diameter and 10 mm high were uniaxially compressed at a rete 1 mm/min. Both tensile and compressive tests were performed along to the extrusion direction.

### 2.2. In Vitro Corrosion

The corrosion behaviour was studied in simulated body fluid (SBF27) prepared according to the Müller, et al. [45]. Both immersion tests and electrochemical potentiodynamic measurements were performed to assess corrosion resistance. In the former, valves 6 mm in diameter and 15 mm in thickness were immersed in SBF for 14 days at 37 °C. Afterwards, the corrosion products were removed by chemical agents according to ISO 8407. For the Zn-based alloys, these products were dissolved in a solution containing 200 g/L CrO_3_. For the Mg–4Y–3RE alloy a solution of 200 g/L CrO_3_, 10 g/L AgNO_3_ and 20 g/L Ba(NO_3_)_2_ was used for this purpose. The corrosion rates were then calculated in mm/year using the weight losses measured on a balance with an accuracy of 0.1 mg, according to ASTM G31-72. The potentiodynamic curves of the alloys were measured in SBF at 37 °C (Gamry FAS1 potentiostat, Warminster, PA, USA). Experiments were performed in a standard three-electrode set-up: with the sample (a coupon 6 mm in diameter and 2 mm in thickness) as the working electrode, two graphite rods as the counter electrodes and Ag/AgCl/KCl (3 mol/L) as the reference electrode (SSCE) with a potential of 0.199 V/SHE. All potentials presented in this paper were measured against SSCE. Potentiodynamic curves were scanned from −0.2 V/Eocp to +0.5 V/Eocp at a rate of 2 mV/s. Before the measurement, the samples were stabilized in the solution for 30 min.

### 2.3. In Vivo Corrosion

**Figure 2:**
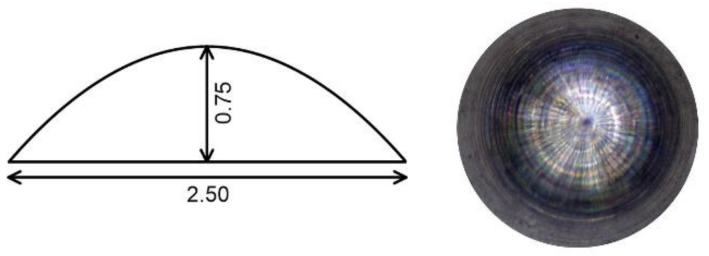
Hemispherical implants used in *in-vivo* animal testing. All dimensions are in mm.

**Figure 3:**
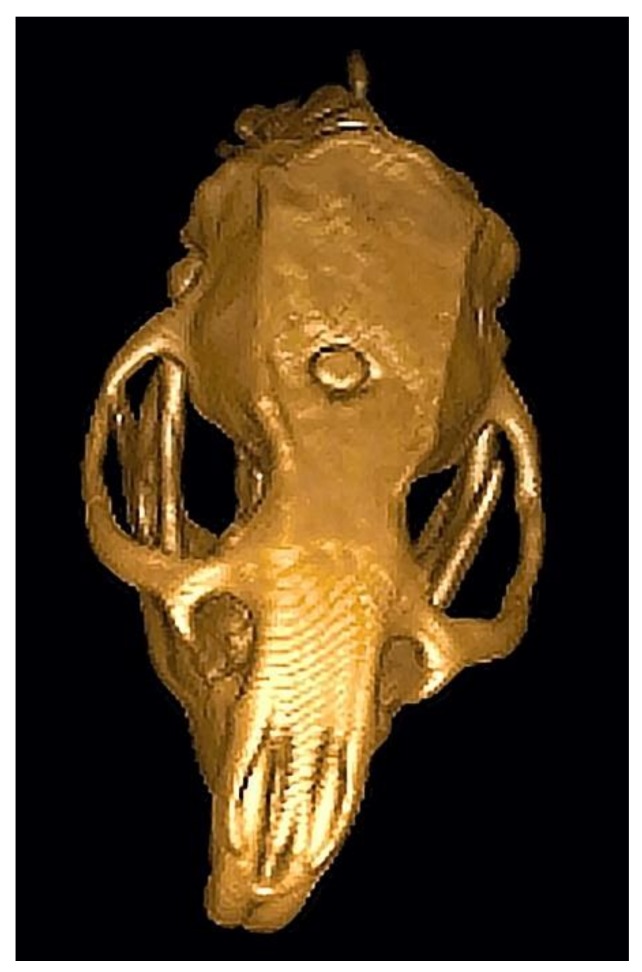
Computed tomography (CT) image of the rat’s cranium with the biodegradable implant.

For in vivo animal tests, the alloys were machined to hemispherical implants of 2.5 mm in diameter, see Figure 2. Ten male Wistar rats (Velaz, Prague, Czech Republic) were used as an experimental model. The animals were divided into two identical groups, one received the Mg-4Y–3RE implants and the other received the Zn-2Mg implants. The implants underwent sterilization by steam autoclaving before implantation. The surgical procedure was conducted according to the university animal ethics committee. The animals were anaesthetized with 3% isoflurane (flow 300 mL/min) applied via face mask. After anaesthesia, the rats were placed into prone position and incision of the skin, subcutaneous soft tissues and periosteum were made. A hole of 2.5 mm in diameter was drilled into the bone between bregma and lambda with the use of a conventional device (W&H, SI-923, Austria, small diamond round bur, 10,000 rpm). One implant was placed into the bone defect in each rat (Figure 3). The bone cavity with the implant was then covered with periosteum, subcutaneous soft tissues and skin, sutured in layers with monofil. The rats were placed into each own cage and observed until completely recovered from anaesthesia. Postoperatively all rats were allowed to move freely, and each rat was housed alone in the cage to minimize postoperative damage of the wound. Rats were tested to evaluate motor and behavioural functions and CBCT (Cone Beam Computed Tomography) was used to observe possible gas formation. After a 12-week interval following implantation, the rats were euthanized, the implants were carefully extracted and subsequently analyzed. The corrosion products were removed firstly mechanically and subsequently by a water solution of 200 g/L CrO_3_, according to ISO 8407, and the corrosion rates were calculated from the weight losses, according to ASTM G31-72. The surface of corroded samples was analyzed using X-ray diffraction (XRD) (X-Pert Pro). To determine the chemical and phase compositions of corrosion products and assess the corrosion mechanisms of the alloys, the in vivo corroded samples were examined in cross-sections by a scanning electron microscope (SEM) Tescan Vega 3 and energy-dispersive spectrometer (EDS).

## 3. Results

### 3.1. Microstructure and Mechanical Properties

**Figure 4:**
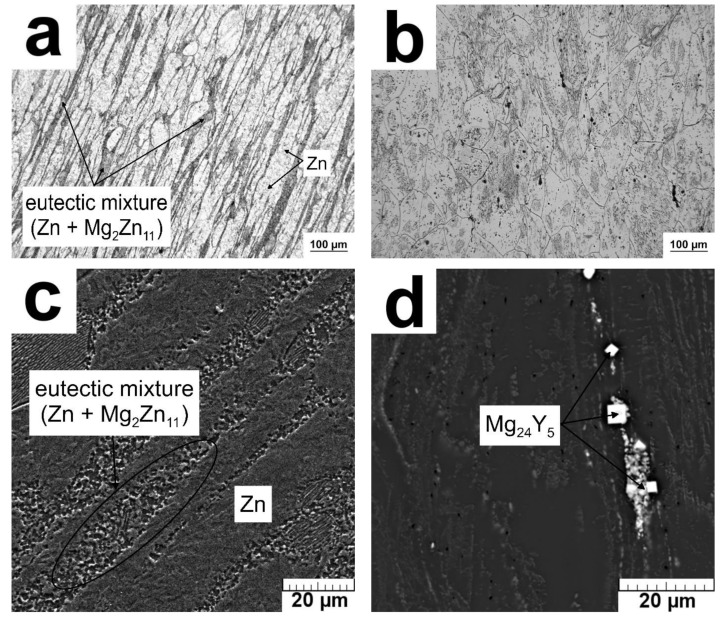
Microstructures of the examined alloys: (**a**) Zn–2Mg (OM), (**b**) Mg–4Y–3RE (OM), (**c**) Zn–2Mg (scanning electron miroscope (SEM)), (**d**) Mg–4Y–3RE (SEM).

Microstructures and mechanical properties of the alloys are briefly summarized in Figure 4 and Table 2. The Zn–2Mg alloy is composed of dynamically recrystallized Zn phase with equiaxed grains of 10 µm in size (measured on 100 grains by the line intercept method) appearing as light regions in Figure 4a. In this figure, there are also Mg_2_Zn_11_ intermetallic phases (dark) arranged parallel to the hot extrusion direction. The morphology of eutectic particles is better seen on an SEM view of the etched sample in Figure 4c. It is obvious that eutectic lamellae typical for the as-cast state [49] are broken down and the eutectic mixture slightly coarsened during hot extrusion at a relatively high temperature. 

**Figure 5:**
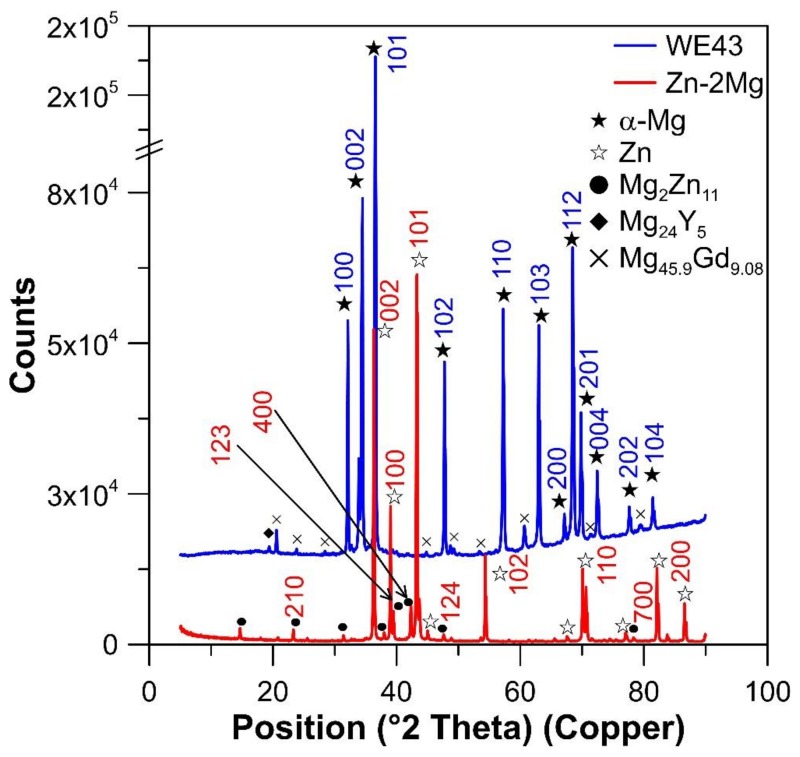
X-ray diffraction (XRD) of Zn-2Mg and Mg-4Y-3RE. Measured area is parallel to the extrusion direction. Subsequent powder diffraction patterns’ ID have been used: Mg (00-001-1141), Mg_24_Y_5_ (00-016-0854), Gd_9.08_ Mg_45.9_ (01-071-8824), Zn (00-001-1238), Mg_2_Zn_11_ (04-007-1412). Numbers above the symbols reflect diffraction peaks of specific planes.

The Mg–4Y–3RE alloy contains recrystallized slightly elongated grains of primary Mg in which major fractions of Y (3.8 wt %) and RE (3.2 wt %) are dissolved. The majority of intermetallic phases such as Mg_14_Nd_2_Y was dissolved during heat treatment at 525 °C/8 h. Still, some Y-enriched Mg_24_Y_5_ phase and Mg_45.9_Gd_9.08_ were presented in the material based on the XRD results (Figure 5). This phase was observed also in literature [55] and its stoichiometry is close to Mg_5_Gd [56,57]. Presented particles were arranged occasionally in the rows parallel to the extrusion direction (Figure 4d).

**Figure 6:**
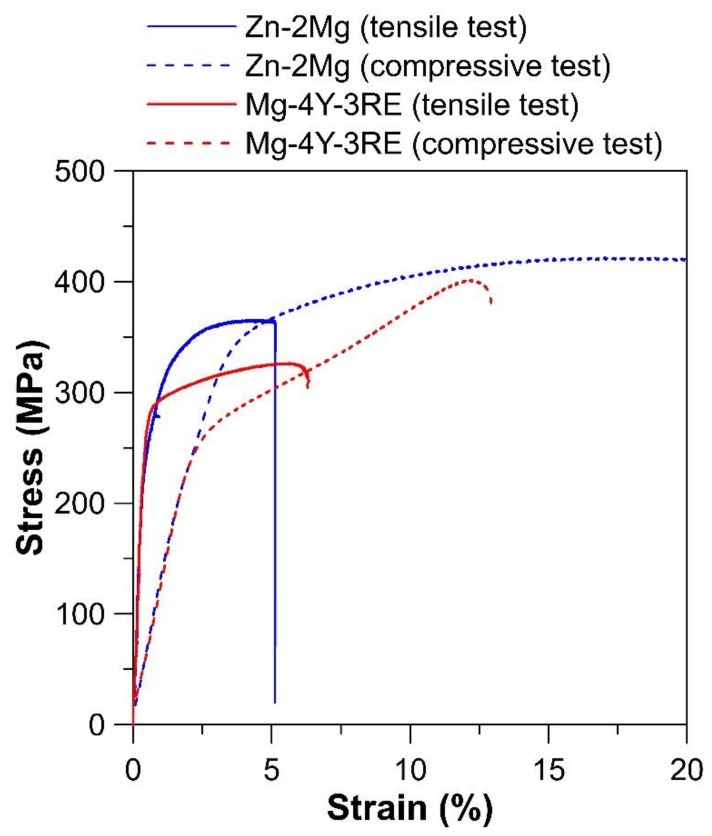
Stress–strain curves of Zn–2Mg and Mg–4Y–3RE alloys for compression and tensile loading.

The stress–strain curves of studied materials both for compression and also tension loading are displayed in Figure 6. The values of mechanical properties are more clearly evident in Table 2. The existence of common fibre texture of the material with a hexagonal close-packed structure related to the extrusion with the majority of basal planes oriented parallel to the extrusion direction significantly affects the tensile yield strength (TYS) and compressive yield strength (CYS) values for tensile loading in the extrusion direction [58,59,60,61]. During tensile testing, materials are oriented inappropriately for basal slip and twinning and, therefore, higher stresses are necessary to activate more complicated slip and twinning mechanisms. As a consequence, the material seems to be more strengthened. However, if the same material is subjected to compressive tests in the direction of extrusion, the orientation is suitable for twinning and, therefore, the value of CYS is decreased compared to the value of TYS [58,59,60,61]. Based on the observed week texture of Zn–2Mg alloy, values of CYS and TYS are very close and any significant anisotropy of mechanical properties has been observed. By contrast, a slight difference is evident for CYS and UTS in the case of Mg-4Y-3RE extruded alloy. However, the difference of 30 MPa is quite small and this suggests that texture strength is not strong. This is often observed in the case of magnesium-based alloys with RE elements in the structure because these elements suppress the formation of strong basal texture [61,62].

Table 2 shows that the Zn–2Mg alloy exhibits a slightly lower hardness and yield strength, but a higher ultimate strength, than the Mg-based counterpart. However, it should be noted that both alloys fulfil the general mechanical requirements for metallic biodegradable implants (TYS > 200 MPa, UTS > 300 MPa) [28].

### 3.2. In Vitro Corrosion Behaviour

**Figure 7:**
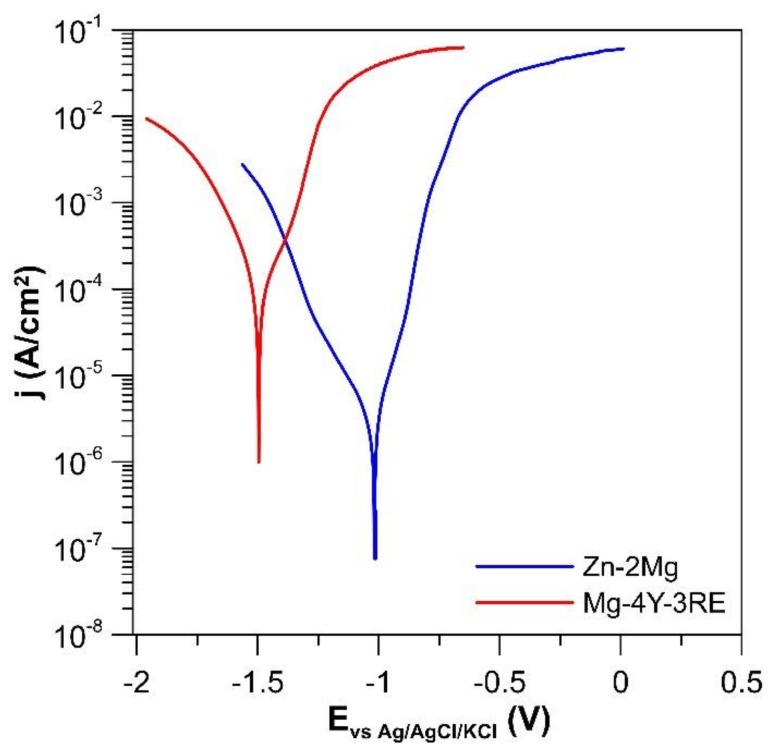
Potentiodynamic curves of the examined alloys in the simulated body fluid (SBF).

Figure 7 shows the potentiodynamic curves of the examined alloys in the SBF and Table 3 summarizes both in vitro electrochemical corrosion parameters and in vitro corrosion rates determined from weight losses of the alloys after exposition in SBF. As was expected, the potentiodynamic curve of the Zn–2Mg alloy is shifted to more noble potentials and lower current densities as compared to the Mg–4Y–3RE alloy (Figure 7). The difference between corrosion potentials of the alloys is 570 mV and that between corrosion current densities approximately one order of magnitude. In vitro corrosion rates of the Zn–2Mg and Mg–4Y–3RE alloys in the SBF calculated by using the Tafel extrapolation are 0.09 and 1.51 mm/year, respectively. The immersion tests confirmed superior corrosion resistance of the Zn–2Mg alloy compared to the Mg–4Y–3RE. Corrosion rates were determined as 0.09 and 0.50 mm/y for Mg–2Zn and Mg–4Y–3RE respectively. It is known that electrochemical methods in many cases provide different results of estimated corrosion rate compared to the classical weight changes measurements or hydrogen release for magnesium alloys. This is also seen in the present case, where the corrosion rate of the Mg–4Y–3RE alloy estimated from weight changes is threefold lower compared to the measurement by Tafel extrapolation. However, the potentiodynamic curves are measured at the beginning of exposure after one hour of stabilization in solution compared to the long immersion test performed for 42 days. The corrosion of magnesium is known to reduce after some time due to the protective character of the corrosion products formed. These products are in many cases composed of magnesium hydroxide, which is still partially soluble and porous. Therefore, deceleration of corrosion rate is in many cases connected with the formation of thicker corrosion product layer on magnesium, which takes some time. By contrast, on the surface of the zinc-based alloy, a thin protective surface passive layer is formed very easily and quickly. Consequently, estimation of corrosion rate from Tafel slopes after 1 h provides the comparable result as weight changes after a significantly prolonged time of exposure.

**Figure 8:**
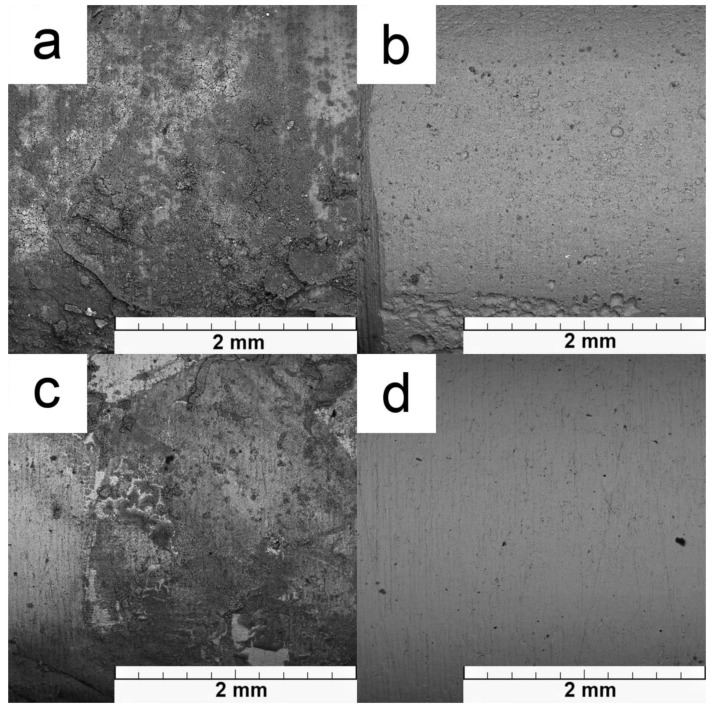
The surface of samples after in-vitro corrosion tests before (**a**,**c**) and after (**b**,**d**) removing of corrosion products: (**a**,**b**) Mg–4Y–3RE, (**c**,**d**) Zn–2Mg.

Surfaces of samples after immersion tests are displayed in Figure 8. Global chemical composition based on EDS analysis is shown in Table 4. Both alloys are covered by corrosion products composed of oxides and phosphates. The corrosion products on magnesium alloy are predominantly composed of magnesium hydroxide, which is precipitated on the surface and creates massive corrosion products. This is supplemented by magnesium doped calcium phosphate. In the case of Zn–2Mg, the surface is enriched by oxygen, phosphorus and calcium, which confirm the precipitation of mixed hydrated oxides, chlorides, phosphates and carbonates. The bigger difference between both studied materials is evident after removing corrosion products (Figure 8b,d). Magnesium alloy is in this case locally attacked. On the contrary, the surface of zinc alloy is attacked faintly compared to the Mg–4Y–3RE. This behaviour clearly illustrates the observed differences in corrosion rates.

### 3.3. In Vivo Corrosion and Biocompatibility

Although the biocompatibility assessment was not the main task of this study, visual observation of the living animals and inspection of implant location 12 weeks after implantation was performed. These tests show that the biodegradable Zn and Mg-based implants have no adverse effects on the behaviour or physical condition of rats. Moreover, gas bubbles and inflammatory reaction of the living tissue were not detected after 12 weeks period.

**Figure 9:**
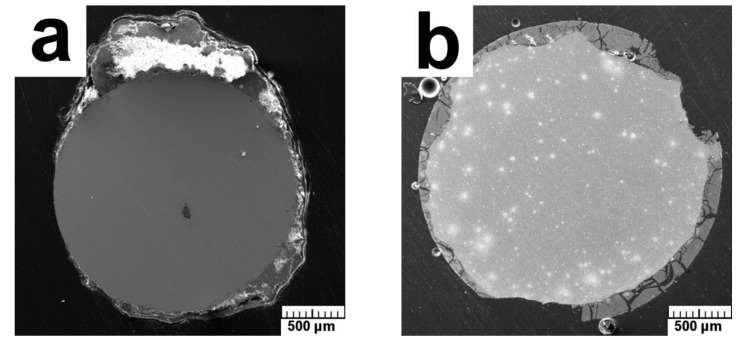
The overall views of the cross-sectioned samples after 12-week in vivo corrosion: (**a**) the Zn–2Mg alloy, (**b**) the Mg–4Y–3RE alloy.

Based on the weight changes, in vivo corrosion rates were established as 0.10 and 0.90 mm/year for the Zn–2Mg and Mg–4Y–3RE alloys, respectively. This is in a relatively good agreement with the in-vitro tests. The overall views of the cross-sectioned samples after 12-week in vivo corrosion are shown in Figure 9. It is observed in Figure 9a that the Zn–2Mg alloy retains the round shape after in-vivo exposition suggesting relatively slow and uniform corrosion. In contrast, the reference Mg–4Y–3RE alloy (Figure 8b) shows an irregular shape and thicker corrosion products confirming a higher in vivo corrosion rate of this alloy.

**Figure 10:**
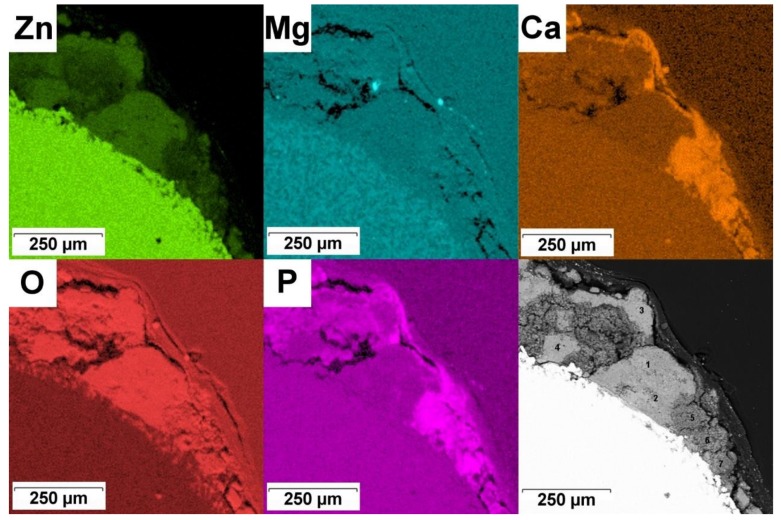
Corrosion products on the in-vivo corroded Zn–2Mg alloy and corresponding elemental maps.

X-ray elemental mapping and point EDS microanalysis (except for C) were performed in selected regions to study corrosion products covering the alloys. Figure 10 and Table 5 characterize the Zn–2Mg alloy. It is obvious that corrosion products consist of at least two constituents: The first one (lighter in Figure 10) has higher Zn and O concentrations and lower P and Ca concentrations (points 1–4 in Table 5). The second component of the corrosion layer (darker in Figure 10) contains, besides Zn and O, enhanced concentrations of Ca and P (points 5–7 in Table 5). Besides, traces of Cl are detected in corrosion products covering the zinc alloy. It can be assumed that the first component of the corrosion layer is dominated by ZnO (or a mixture of ZnO + ZnCO_3_) while the second contains a mixed calcium zinc phosphate (Zn, Ca)_3_(PO_4_)_2_.

**Figure 11:**
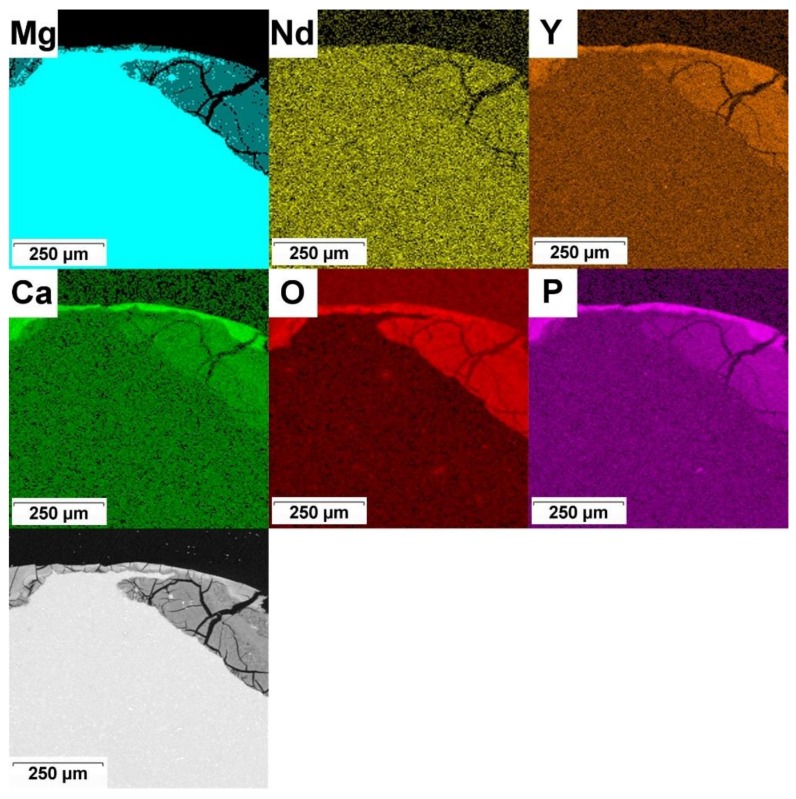
Corrosion products on the in vivo corroded Mg–4Y–3RE alloy and corresponding elemental maps.

Elemental composition of corrosion products on the Mg alloy is presented in X-ray maps in Figure 11. As in the previous case, corrosion products mainly contain Mg, O, P and Ca (light carbon was not measured). These elements are not distributed uniformly as P and Ca are enriched in external parts of the corrosion layer. The main corrosion products of the Mg–4Y–3RE alloy are thus Mg(OH)_2_, MgCO_3_ and (Mg, Ca)_3_(PO_4_)_2_ which is in accordance with observations of other authors [53,63,64]. Rare earth metals partially substitute magnesium and calcium in these compounds.

## 4. Discussion

Tensile mechanical properties of extruded Zn–2Mg alloy were determined as 235 MPa for TYS and 265 MPa for UTS. These values are significantly higher compared to the 200 and 270 MPa determined in the case of extruded Zn–1Mg alloy by Li et al. [33]. Gong et al. [32] measured mechanical properties of extruded Zn–1Mg and achieved the values about 180 MPa for TYS and 250 MPa for UTS with elongation of 10%. Increased mechanical properties in the present study are related especially to the increased amount of Mg in the alloy and, therefore, strengthening by secondary phases which are presented in the microstructure. Strengthening by grain boundaries described by the Hall–Petch relation is another well-known strengthening mechanism. However, the grain size of all compared materials was close to 10 µm. A solution strengthening mechanism is not considered because solid solubility of Mg in Zn is negligible and the texture contribution effect is considered to be very low in the present state due to the low texture strength. Regarding mechanical properties, modulus of elasticity has an important role especially for applications like fixation devices. The low value of modulus of elasticity of magnesium-based materials (≈45 GPa) is relatively close to the general value of natural bones (≈20–25 GPa), which partially suppress the stress-shielding effect. This effect is connected to the non-uniform distribution of loading on implant and healing bone. The rough value of modulus of elasticity (≈90 MPa) obtained for Zn–2Mg from tensile curve indicate that combining these materials with bone may lead to greater susceptibility to the stress shielding effect.

Corrosion behaviour of this alloy was compared with well-known biodegradable Mg-based alloy Mg–4Y–3RE. Both measurements have shown that zinc alloy corrodes relatively slowly and uniformly. Comparing the corrosion rates of Zn–2Mg and Mg–4Y–3RE calculated from weight losses, it can be seen easily that Zn–2Mg alloy degrades in in vitro and in vivo conditions 5 or 9 times slower, respectively. This is due to several differences in the corrosion behaviour of zinc and magnesium.

For Mg, anodic and cathodic reactions following can be written [65]:

Mg → Mg^2+^ + 2e^−^

H_2_O + 2e^−^ → H_2_ + 2OH^−^

Corrosion mechanism for Zn can be described as [65]:

2Zn → 2Zn^2+^ + 4e^−^

2H_2_O + O_2_ + 4e^−^ → 4OH^−^

While magnesium is not sensitive to the oxygen present in the corrosion medium, the cathodic reaction for zinc involves the reduction of oxygen. Therefore, there is nearly no evolution of hydrogen during zinc corrosion. This is important not only in terms of corrosion rate but also in terms of the healing process.

Released cations and OH^−^ anions take place in reactions with different components of the medium in which the samples are exposed and form corrosion products. In the case of Mg–4Y–3RE, the considered corrosion products were Mg(OH)_2_, (Mg, Ca)_3_(PO_4_)_2_ and MgCO_3_. Formation of these compounds is depicted by the following equations:

Mg^2+^ + 2OH^−^ → Mg(OH)^2^

3Mg^2+^ (Ca^2+^) + 2HPO_4_^2−^ + 2OH^−^ → (Mg,Ca)_3_(PO_4_)_2_ (insoluble) + 2H_2_O

Mg^2+^ + HCO^3−^ + OH^−^ → MgCO_3_ + H_2_O

**Figure 12:**
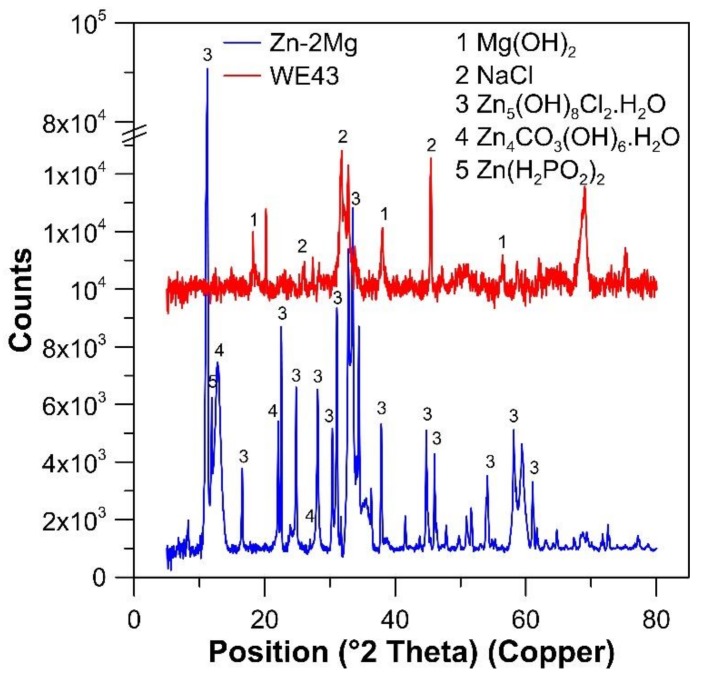
X-ray diffraction of the surfaces of Zn–2Mg and Mg–4Y–3RE after in vivo tests. Subsequent powder diffraction pattern IDs have been used: Mg(OH)_2_ (00-001-1169), NaCl (00-001-0993), Zn_5_(OH)_8_C_l2_·H_2_O·(00-007-0155), Zn_4_CO_3_(OH)_6_·H_2_O·(00-003-0787), Zn(H_2_PO_2_)_2_·(04-010-4355).

Based on the XRD measurements (Figure 12) of the corrosion product on samples from in vivo tests, only Mg(OH)_2_ was formed as the main component of corrosion products. The presence of NaCl in Figure 12 is not related to the formation of this product during the in vivo test. Instead, it was crystallized from residual solution probably in some crack between corrosion products after removing the sample from animal, washing the sample and drying it. The same information can be obtained from Figure 11, where huge areas containing Mg, O and partially Y are evident. There also exists a thin layer of calcium, magnesium mixture phosphate, which, due to its low thickness, could not be identified by XRD (Figure 12). It seems that during the corrosion process, pH at the surface is increased early and magnesium calcium phosphates precipitate at the surface. This layer forms a barrier but still the corrosion process continues under it to form predominantly Mg(OH)_2_ or MgCO_3_.

In the corrosion layer on Zn–2Mg samples, ZnO or its hydrated forms, mixed calcium-zinc phosphate and also zinc carbonate are formed. Although EDS microanalysis does not provide accurate values for C, the aforementioned products were confirmed after the immersion test by XRD (Figure 12).

Reactions of zinc cation with the surrounding medium yielding corrosion products are:

Zn^2+^ + 2 OH^−^ →ZnO + H_2_O

3Zn^2+^ (Ca^2+^) + 2 HPO_4_^2−^ + 2OH^−^ → (Zn,Ca)_3_(PO_4_)_2_ (insoluble)+ 2H_2_O

Zn^2+^ + HCO^3−^ + OH^−^ → ZnCO_3_ + H_2_O 

Although both Zn and Ca partially occupy the surface phosphate layer, the dominant cation is Ca^2+^. Therefore, it seems that zinc-based corrosion products are especially carbonates and mixed hydrated oxides and chlorides (Figure 12). Standard potentials of Mg and Zn are −2.372 and −0.762 V, respectively [36], which are very distinctive values. Slower corrosion concerning Zn-based alloys as more noble material is thus partially connected with higher corrosion potential (−0.98 V vs. −1.55 V for Mg–4Y–3RE, Figure 7, Table 3). Also, high hydrogen overpotential of Zn has an important influence on the corrosion rate. Due to the high overpotential, the evolution of hydrogen on the surface of zinc is suppressed. Consequently, the cathodic reaction is dominated by the reduction of dissolved oxygen and this process is controlled by diffusion, which leads to a limitation of cathodic current. This phenomenon was affirmed by measurement of potentiodynamic curves.

Although the differences in the corrosion rate of magnesium alloy in vivo and in vitro are evident and this observation is very well-known, there is a very good similarity between the corrosion rates of the Zn–2Mg alloy in both conditions. Also, the corrosion products were composed of the same constituents, however, it is necessary to mention, that participation of some organic elements in the corrosion process in-vivo is not considered which can be the reason for increased corrosion rate of Mg–4Y–3RE.

The in vitro corrosion rate of the Zn–2Mg alloy has been also determined in other papers [32,33,35]. Gong et al. [32] determined the corrosion rate of the Zn–1Mg alloy in SBF as 0.12 mm/y, which is very close to the observed values. Li et al. [33] calculated corrosion rates of Zn–1Mg immersed in Hank´s solution as 0.15 mm/y from Tafel slopes and 0.08 mm/y from weight changes. This is also close in value to the 0.09 mm/y assessed in our case. Some difference can be connected with the choice of different modelling environment for the immersion tests.

Our earlier studies showed that corrosion rates of Zn–Mg alloy with 0.5–2 wt % of Mg are a very similar and only slight trend in the improvement of corrosion resistance with the increased amount of Mg has been observed [35,49]. This can be also the reason for the slightly decreased corrosion rate of the presented Zn–2Mg alloy that was observed.

The corrosion rate of Zn–2Mg alloy estimated from in vivo tests was determined as 0.1 mm/year. Li et al. [33] determined the in vivo corrosion rate of Zn–1Mg alloy by micro-CT analysis as 0.17 mm/y. Some difference in corrosion rates can be connected with different methodology but both these values are very promising. To establish if the requirements for biocompatibility are accommodated, one can visualize a real application, such as a screw for bone fixation. The bone screw is often fabricated with average dimensions about 30 mm in length and 3 mm in diameter. By simplified calculation, with an assumption of uniform corrosion at a rate of 0.1 mm/year, one obtains that 1 mg of Zn per day is released into the organism. Such an amount is significantly lower than the upper recommended daily limit representing 40 mg of Zn. This implies that the use of zinc alloyed by a small percentage of magnesium remains non-toxic during its use in the form of a small bone-fixation device.

Apart from biocompatibility, biodegradable materials for load-bearing applications also require feasible mechanical performance during the healing process. From the surgical point of view, a fixation screw should preserve at least 95% of its original load-bearing capability during the first 6 weeks after implantation. This means that the screw can undergo such corrosion so that its cross-section would reduce by less than 5%. This requirement gives a maximum acceptable corrosion rate of 0.4 mm/year. Therefore, it can be seen that in the case of the investigated Zn–2Mg alloy, this requirement is satisfied. For the reference Mg–4Y–3RE alloy, the corrosion rate of 0.9 mm/year already exceeds this limit.

## 5. Conclusions

The present paper deals with the study of the microstructure, mechanical and corrosion behaviour of Zn–2Mg and Mg–4Y–3RE alloys. Yield strength and ultimate strength of Zn–2Mg in tension and compression exceed 230 and 360 MPa, respectively, which are considered as sufficient values to ensure mechanical support for healed bone during the regeneration process. Moreover, uniform degradation and low corrosion rates of Zn–2Mg determined both in vitro (0.09 mm/year) and in vivo (0.10 mm/year) fulfil general requirements to ensure sufficient mechanical integrity and low doses of released ions into the human organism during implant degradation. Good biocompatibility of Zn–2Mg was stated based on no adverse effects on the behaviour or physical condition of rats and no inflammatory reaction of the living tissue observed after 12 weeks period of in vivo tests. All these findings demonstrate that the zinc-based alloy Zn–2Mg prepared by conventional casting and hot extrusion process meet the key requirements for load-bearing biodegradable implant application like fixation devices (screws and plates). Although yield strength and ultimate strength of Zn–2Mg and Mg–4Y–3RE are almost similar, the in vivo corrosion rate of Zn–2Mg is nine times lower and the biodegradation is not accompanied by hydrogen release. However, such an advantage over Mg–4Y–3RE is partially negated by low ductility (≈5%) and a higher value of modulus of elasticity (≈90 GPa). Therefore, none of the studied materials is superior and individual alloys may be preferred for specific conditions.

## Figures and Tables

**Table 1:** Chemical compositions of the alloys (in wt %).

Alloy Designation	Element (in wt %)						
	Mg	Zn	Fe	Gd	Nd	Y	Zr
Zn–2Mg	1.6	bal.	0.1	-	-	-	-
Mg–4Y–3RE	bal.	-	-	-	2.8	4.2	0.4

**Table 2:** Mechanical properties of the examined alloys (HV—Vickers Hardness, TYS—tensile yield strength at 0.2% offset, UTS—ultimate tensile strength, E—elongation, CYS—compressive yield strength at 0.2% offset, UCS—ultimate compressive strength).

Alloy	HV	TYS (MPa)	UTS (MPa)	E (%)	CYS (MPa)	UCS (MPa)
Zn–2Mg	97	235	365	4.9	231	426
Mg–4Y–3RE	114	280	316	5.3	239	402

**Table 3:** Electrochemical corrosion parameters (corrosion potential E_cor_, corrosion current density j_cor_) and corrosion rates v_cor_ of the examined alloys.

	*In Vitro* Tests			
Alloy	E_cor_ (mV/SSCE)	j_cor_ (A/cm^2^)	v_cor__PD Curves (mm/year)	v_cor__Weight Changes (mm/year)
**Zn–2Mg**	−980	6 × 10^−6^	0.09	0.091 ± 0.018
**Mg–4Y–3RE**	−1550	7 × 10^−5^	1.51	0.495 ± 0.128

**Table 4:** Chemical composition (in wt %) of corrosion products after immersion tests in SBF.

Alloy Designation	O	Mg	Zn	P	Ca	Y	Nd	Gd	Dy
Mg–4Y–3RE	43.3	48.2	-	3.2	3.6	1.3	0.4	-	-
Zn–2Mg	23.1	0.3	55.9	10.7	10.3	-	-	-	-

**Table 5:** Chemical compositions (in wt %) of corrosion products at points depicted in Figure 9 (C is not included).

Point	O	Mg	P	Cl	Ca	Zn
1	20.8	-	0.4	0.6	-	78.2
2	23.9	-	0.3	0.3	0.3	75.2
3	19.3	-	0.5	1.2	-	79.0
4	21.3	-	-	1.0	-	77.7
5	28.9	0.4	12.9	0.6	11.9	45.3
6	18.9	0.3	8.9	0.4	10.2	61.3
7	35.3	0.6	10.6	0.2	8.2	45.1
